# Measuring health-related quality of life in patients with rare disease

**DOI:** 10.1186/s41687-021-00336-8

**Published:** 2021-07-20

**Authors:** William R. Lenderking, Milena Anatchkova, Robin Pokrzywinski, Anne Skalicky, Mona L. Martin, Heather Gelhorn

**Affiliations:** 1 Patient-centered Research, Evidera, 500 Totten Pond Rd. Fifth Floor, Waltham, MA 02451 USA; 2 Patient-centered Research, Evidera, Bethesda, MD USA; 3 Patient-centered Research, Evidera, Seattle, WA USA

**Keywords:** Rare disease, HRQoL, Clinical outcome assessment (COA), Item banks, Measure adaptation, FDA, Embedded interviews, Disease-specific, Multi-dimensional responder index

## Abstract

**Background:**

There has been a growing emphasis on health-related quality of life (HRQoL) as an important outcome in rare disease drug development, although its assessment may be useful outside the drug development context, including in clinical applications or natural history studies. Central to assessing quality of life in health research is utilizing outcome measures that capture symptoms and impacts of the disease and treatment that are important and relevant to patients. Identifying and implementing valid and reliable tools to measure HRQoL in rare diseases poses unique challenges that often require creative solutions.

**Main body:**

In this commentary, we explore some of the challenges in HRQoL assessment in rare disease, propose solutions, and consider regulatory issues. Some of the solutions discussed entail the use of item banks, adapting existing measures from phenotypically similar disease contexts, use of multi-domain measurement indices, and adapting methods for assessing content validity of existing measures. Current regulatory considerations are discussed and resources outlined.

**Conclusion:**

Quality of life may be the most important endpoint for patients with rare diseases, and the challenges of valid assessment require effort and innovative thinking specific to each context to improve measurement and clinical outcomes.

## Background

In January 2019, the United States (US) Food and Drug Administration (FDA) published revised guidance on rare disease (Rare Disease: Common Issues in Drug Development, January 2019 [1], replacing the August 2015 version). Rare diseases were defined in the Orphan Drug Act (ODA) of 2010 as diseases that affect fewer than 200,000 patients in the US, although many afflict far fewer patients, and they have become increasingly important targets for new drug development. Health-related quality of life (HRQoL) can be an important outcome in rare diseases, due to their chronic nature and often lack of available treatments. Several factors contribute not only to the difficulty of bringing new drugs to market for these diseases, but also to the challenge of assessing HRQoL, including: 1) they are predominantly pediatric diseases (75%); 2) by definition, very small sample sizes are available to study; and 3) they may be characterized by unique symptom clusters or symptoms that mimic other diseases, making diagnosis difficult. While traditional methodologies for demonstrating content validity may sometimes be feasible, psychometric assessments are particularly challenging. Furthermore, in the case of ultra-rare diseases, where there may be fewer than 500 known cases worldwide, it is unlikely to be cost-effective to develop a disease-specific measure or to enroll patients in instrument validation studies of any size. An excellent introduction to the general issues for outcomes researchers in rare disease can be found in the International Society for Pharmacoeconomics and Outcomes Research (ISPOR) Clinical Outcome Assessment (COA) Good Practices Task Force Report on Rare Diseases [2].

## Main text

### Challenges of HRQoL assessment in rare disease

Even though it is difficult to measure HRQoL in rare disease, this does not mean it should not be done. Improving HRQoL may be the most important outcome in many diseases with a chronic course and without cure. HRQoL is often assessed by self-report using patient-reported outcome (PRO) measures. However, with children and in the light of possible cognitive, speech, or motor dysfunction, traditional self-report may not be useful, and the use of specialized reporting strategies or a caregiver-completed observer-reported outcome (ObsRO) measure may be required. Rare disease may impact multiple organ systems, cause permanent damage to the body, or have a highly heterogeneous presentation, making meaningful treatment benefit unclear in the context of assessment of drugs and biomedical treatments. Accordingly, some researchers may choose to emphasize one or two domains—out of several—where measurement strategy and treatment benefit may be clearer. The natural history studies called for by the FDA in its guidance may not have been completed yet, contributing to an incomplete understanding of rare diseases and their course. Understanding of rare diseases may change rapidly, even during a clinical trial or natural history study, due to new treatments or discoveries about the illness. Pediatric growth and development are challenges for longitudinal assessments. The traditional drug development approach and timeline can be altered considerably (e.g., with a natural history study being transformed into a phase II study). Disease-specific measures often do not exist, while generic measures may be too broad, and traditional guidelines for statistical validation are strongly challenged by very small samples. Factors such as risk and benefit if alternative treatments or new treatment technologies are available may also play a role in the evaluation of treatment benefit.

Some of these challenges are illustrated by WHIM syndrome (warts, hypogammaglobulinemia, infections, myelokathexis), an ultra-rare primary immunodeficiency [3], of which there have been fewer than 200 cases reported worldwide since its discovery in 1964. This disease can present with numerous and uncontrollable warts all over the body, including venereal warts and their associated risk of malignant complications; upper respiratory infections, including pneumonia, abscesses, bronchitis, and/or sinusitis; and retention of cells in the bone marrow (myelokathexis). Due to the various complications that are possible, and the range of severity, it is difficult to know how to measure meaningful benefit and how to weigh improvements in warts (which can be so severe they may impact the ability to walk), which may improve day-to-day living considerably, against prevention of episodic infections that may be life-threatening. Furthermore, there is no disease-specific measure that can capture the variety of possible outcomes in this condition, nor would it be a good use of patient resources to validate a new instrument given the small disease population.

Clinical input is a way to provide meaningful insights into rare disease characteristics, outcomes of interest, and interventions. However, individual clinicians may have limited opportunities to observe firsthand the signs of disease in clinical practice. Rare diseases that impact cognition may also alter the patients’ insight into their symptoms, further limiting the clinician’s ability to understand them.

Regarding statistical validation, the rules of thumb of *n* = 30 for stable evaluation of means under the central limit theorem, or *n* = 10 observations per item for factor analysis or 10 observations per variable in a regression model, simply are impossible to achieve for many ultra-rare diseases.

### Proposed solutions

Solutions to the problems outlined above can require considerable creativity as evidenced by recently applied methods.

#### Use of item banks

One potential approach to addressing the challenges of rare disease PRO measure development and validation involves harnessing the power of existing item banks to develop PROs that are appropriate for the desired context of use and can be developed relatively rapidly. There are several existing and available item banks that cover a range of HRQoL concepts: the Patient Reported Outcome Measurement System (PROMIS) [4], Quality of Life in Neurological Disorders (Neuro-QoL) [5], and the European Organisation for Research and Treatment of Cancer (EORTC) [6]. These item banks can be used to develop measures specifically suited to the measurement context, including targeting specific domains or concepts and modules for specific disease sub-types, for adults or pediatric populations and are suitable for various modes of administration. This approach has been successfully demonstrated in a prior program of research on tenosynovial giant cell tumors (TGCT), wherein the PROMIS [4] physical functioning (PF) item bank was used to develop custom patient-reported outcome measures [7].

In the application of PROMIS item banks to measuring outcomes in TGCT, significant obstacles were encountered due to the rare nature of the disease, limited prior research, heterogeneity in the types of physical impacts due to varied location of the tumors in each patient, and a large amount of variability in the severity of impacts across patients. The program of research included gathering direct patient input through interviews, patient-completed symptom and impact checklists, clinical expert input, and leveraging item information from PROMIS item banks of relevant concepts using item response theory (IRT) parameters [7, 8]. Through this approach, the team identified relevant symptom and impact concepts and subsequently selected statistically efficient items (i.e., non-overlapping conceptually and in terms of severity on the latent trait) from the PROMIS item bank for inclusion in custom short forms. These custom short forms allowed for heterogeneity in physical functioning impacts by varying the items that were presented to participants based on the location of their tumor. The custom forms were also designed to assess a wide range of impact severity, allowing for the measurement of changes in physical functioning that patients had experienced over the course of the trial, regardless of whether their impacts were mild or severe.

The PROMIS PF custom forms simultaneously facilitated the measurement of the construct of interest on a single latent physical functioning scale, while also allowing for an analysis of physical functioning as an outcome in the full sample, regardless of the tumor location or extent of the impact [8]. This is because PROMIS item bank domains are comprised of groups of items that are of high quality and calibrated as a set to yield established statistical properties. The candidate items have often been thoroughly validated and tested across a range of populations. The PROMIS PF custom short forms were validated [9] and included in a phase III clinical trial of patients with TGCT, which demonstrated significantly better improvements in physical functioning among patients receiving the treatment versus placebo [10]. This approach capitalized on the availability of a free-to-use, high-quality item bank that covers a range of constructs, and efficiently leveraged the limited time and patient population to develop a valid and psychometrically sound PRO measure of the concept of interest. The approach described above is likely to be relevant for many rare disease applications.

#### Adapting existing measures

Another approach to measurement in rare diseases is to adapt existing measures from a phenotypically similar disease to the rare disease context. For example, in one set of studies, the Unified Parkinson’s Disease Rating Scale (UPDRS)-2, originally developed for and widely used in Parkinson’s disease (PD), was adapted for use in pantothenate kinase-associated neurodegeneration (PKAN), a rare disease causing similar motor and speech deficits although with a completely different etiology [11]. In this instance, the UPDRS-2 was adapted by a group of clinical experts in PKAN, and the adapted version was tested for content validity and test-retest reliability in a small sample of cognitive interviews that were repeated 2 weeks later. This study also served as a natural history study [12, 13].

#### Multi-domain responder index

Another approach to measurement could be the use of a multi-domain responder index (MDRI), which the FDA has discussed in its guidance on multiplicity [14] as a way to handle heterogeneity and multiple domains of interest within a disease population. The fundamental concept of an MDRI is that each relevant domain in the condition is measured by a valid instrument (would have to been developed in another condition in most cases) that has a responder definition that is pre-identified—in other words, a within-patient change that is meaningful is pre-specified for several domains. For example, considering WHIM syndrome, an MDRI might assess meaningful changes in warts, infection rates, and improved social functioning. (This is hypothetical as a responder definition [RD] has not been established on any instrument for warts to our knowledge.) This is relevant and may be useful where it is important to ensure that patients show change on several different domains, which may be quite different from each other. However, to our knowledge, no medicine has been approved to date based on an MDRI, so it has to still be considered a novel approach. Another potential issue with this approach is that RDs may be specific to a disease context, and therefore the validity of their application to a rare disease might be questioned.

#### Adaptive methods of content validity assessment

In the third patient-focused drug development guidance [15], the FDA reports it will not examine the psychometric properties or quantitative performance of a COA until the content validity is established, specifically, “data related to the instrument’s other measurement properties will not be reviewed by FDA until content validity of the instrument has first been established.” This emphasizes the critical need for content validity of a measure, which can be even more important in rare disease research because of the small samples sizes for assessing measurement properties.

In the context of rare disease, assessing the content validity of COAs may require adaptive methods for data collection, using technology, triangulating methods, or mixed methods (mix of interview, survey). To identify more universally experienced symptoms in rare, heterogenous conditions, concept frequency can be assessed within subgroups of patients based on age, sex, or disease severity, for example, to identify commonly experienced concepts across the continuum of disease severity. Likewise, mixed methods of data collection (e.g., surveys and interviews combined) or different public sources of data collection (i.e. patient advocacy group discussion forums, FDA-led Patient-focused Drug Development public meetings) can be helpful to provide more data to assess concepts important to patients. Additionally, several other methods can be combined to increase evidence for content validity, including triangulation methods (which can be used to ascertain overlap between patient- and observer-report of concepts [16], best-worst scaling, and digital assessments). Alternate approaches to validation have included iterative validation steps in similar indications, as illustrated by Harrington et al. 2019 [17]. For example, in the development of a caregiver impact questionnaire for rare pediatric lysosomal storage diseases, PRO measure developers relied on developing and validating the instrument in three lysosomal disease patient groups to enable timely use in clinical treatment trials [17]. Other approaches for assessing content validity of measures in rare disease may require utilizing diverse sources of patient input on concepts of interest (i.e., transcripts from FDA Voice of Patient Public Meeting, advisory groups, social media, natural disease history, etc.) [18–21].

Engaging rare disease networks, disease registries, or patient advocacy groups in research is important in contributing to development of research plans and materials and identifying and including patient or caregiver participants to assess HRQoL outcomes. Patients with rare disease can be hard to find, and rare disease networks or patient advocacy groups may provide a concentration of patients who are highly motivated and knowledgeable about their disease and willing to participate in research.

Patients, or caregivers of patients, enrolled in clinical trials may be a resource for qualitative data collection. These interviews are sometimes termed, “embedded qualitative interviews” or “exit interviews,” depending on when the interviews take place. Given that it is sometimes difficult to access patients for interviews, those enrolled in a clinical trial are a valuable resource.

If a program is in phase II for example, data may be collected to help inform the phase III pivotal study in terms of endpoint measurement and trial design. Data collected from the embedded interviews can help identify HRQoL questionnaires that may be utilized later in the trial program. Interview data may serve as a foundation for meaningful treatment benefit evidence because the concepts are meaningful to the participants. The concepts, such as symptoms or impacts, may be mapped to existing questionnaires that may be used in future trial phases. If questionnaires have already been identified, the embedded interviews may provide an opportunity to gain insight about the questionnaires themselves and serve to provide evidence of content validity of the questionnaires in a manner that allows for efficient participation of research subjects.

If a trial has progressed and participants are interviewed later in product development, phase III for example, there are other opportunities for patient insight. The trial participants will be able to speak to changes they experienced with the intervention; the way in which they describe their changes in symptoms can be powerful. Often, participants are able to give insight to the timeline of experiencing a change, if the change was sustained, and if the change provided a meaningful treatment benefit. Each sign, symptom, or behavior can be deeply explored in these embedded interviews. The areas of a meaningful treatment benefit with signs, symptoms, behaviors, and impacts can be reported and treatment benefits about the patient’s HRQoL shared.

In embedded interviews, data can be collected about the intervention itself (e.g., mode of delivery, frequency of administration, who may need to administer the intervention), how treatment benefits are described (e.g., changes in severity, frequency, duration), descriptive examples of symptom changes (e.g., illustrative descriptions that could be used in messaging), and insightful examples of changes in daily impacts (e.g., mobility, emotional or changes in mood, perspectives about their future). Other areas of impact can be explored, such as the journey to diagnosis, which is often lengthy and complex for rare conditions, the caregiver burden, unmet medical needs, the patient’s wiliness to take risks with new treatment, treatment preferences, and the way the condition impacts daily life (activities of daily living, relationships with family and friends, social impacts, financial burden, changing needs for routines such as diet, equipment, ancillary treatment, etc.).

In each of the first four patient-focused drug development documents from the FDA [15, 19, 22, 23], exit surveys or interviews are mentioned. The feedback highlights the unique perspectives that can be gained by collecting data directly from the patient (or caregiver) [15, 19, 22]. With rare conditions, clinical guidance and case series are sometimes the only existing sources of patient experience data. Qualitative research interviews should be considered to gain valuable insight from the trial participants. The scope of the interview can be crafted based on the aims of the research and the timing of the data collection.

#### Updating existing content validity evidence may be needed with new advances in treatment

When advances in treatment are significant enough to change the patient experience of their condition and influence HRQoL by emphasizing different concepts of interest than were used in the traditional measurement models, qualitative work may need to be repeated to update evidence of content validity. One such example is the new C1-Inhibitors used for prophylaxis in hereditary angioedema (HAE). Patients on these new treatments are now able to engage with life more, live in less fear and anxiety, and they report changes in severity, frequency, and duration of all HAE symptoms. The focus is no longer solely on the swelling attack. These significant changes in overall patient burden and quality of life warrant re-examination of the work substantiating the content validity of HRQoL assessment to ensure the concept coverage is still complete.

### Regulatory considerations for HRQoL assessment in rare disease

How a concept is addressed in regulatory guidance documents, as well as how often results for a specific concept are included in label claims, can provide an insight into the level of attention devoted to the concept from the regulatory perspective. Two recent reviews examined the use of PRO measures in approved rare disease labels [24, 25] demonstrating low rates of PROs in label claims. The first examined labels of FDA-approved orphan drugs between 2002 and 2017 and revealed that, while the number of orphan drug approvals has continued to rise, only 8.3% of approved labels included results based on PRO measures. Most of these claims were based on symptom measures and only one product with HRQoL labeling was identified [24]. A later review for the same period, including European Medicines Agency and FDA approvals, reviewed 258 designations in rare non-oncology disease. Only 17.4% of reviewed designations included PROs, and HRQoL was rarely assessed [25]. Moreover, less than half of current clinical trials in rare disease include a PRO based on review of data from clinicaltrials.gov. [25]

In addition to considerations mentioned above, the latest draft FDA guidance for rare disease [1] emphasizes the importance of patient centricity in drug development. It suggests encouraging the engagement of patients, caregivers, and advocates in the drug development process and through the inclusion of outcome measures that reflect how a patient feels and functions. The specific challenges associated with the selection of an outcome assessment are noted, but the guidance stops short of offering specific solutions to these challenges. Assessment of HRQoL specifically is not discussed in the guidance, and suggestions are not provided for validation studies in the face of very small samples. The FDA suggests that study design and conduct are critically important (e.g., thorough training of clinical raters, having raters be otherwise independent of trial administration—both steps designed to improve intra- and inter-rater reliability and reduce subjective bias). Effective blinding of treatment assignment is also emphasized. It is possible that outcomes researchers and companies sponsoring drug development in rare disease may be in the difficult position of arguing for the validity of results while lacking evidence meeting historical standards. At times, based on unmet medical need, regulators appear willing to be persuaded by these arguments, but it is possible they may impose post-approval requirements, such as safety registries. The importance of early engagement with the FDA is emphasized and the modification of existing outcome measures is encouraged over the development of new ones.

A more detailed guidance on outcome measure development in rare disease was provided by the ISPOR COA Emerging Good Practices Task Force Report [2]. The report follows the FDA Roadmap to Patient-Focused Outcome Measurement in Clinical Trials [26] and discusses several challenges with an emphasis on modifying existing measures consistent with the FDA rare disease guidance. While this document provides the most in-depth practical guidance for addressing challenges in the selection of outcome measures for rare disease clinical trials, the extent to which these recommendations are accepted by regulatory agencies is unclear. In addition, recent reviews [24, 25] have suggested that stakeholders experience difficulties following the proposed approach, which is also associated with substantial demand on resources.

Overall, there is an increasing interest and emphasis on including the patient perspective in rare disease drug development, but also a wide recognition that specific challenges exist, which require unique, out-of-the box solutions.

An interactive, one-day meeting in 2018 aimed to discuss challenges in developing rare disease regulatory strategies and included 90 leaders with regulatory, biopharmaceutical, and not-for-profit organizations. The meeting report proposed several strategies, including the use of natural history studies, novel approaches to clinical trial design, and novel analytical approaches [27]. Recommendations specific to the development of clinical endpoint measures included using registry data and real-world evidence to develop the evidence base for validity of measures and an emphasis on the importance of collaboration among all stakeholders. The meeting report does not focus specifically on assessment of HRQoL.

An alternative strategy focusing specifically on selection of COAs to assess treatment benefit in rare disease trials has been proposed [25]. The authors suggest that well-validated generic HRQoL measures should be used to describe the disease burden; well-validated measures specific to relevant body function should be used to measure treatment impact and test hypotheses; patients’ perceptions of change should be assessed by study specific diaries, while meaningfulness of change and patients’ perspectives on benefits and risks should be assessed through qualitative interviews in clinical trials. However, the authors do not discuss the extent to which these measures should be tested in the target population to align with FDA recommendations.

While others have noted some challenges and possible solutions for incorporating COA measures in rare disease research, the unique challenges of each patient population persist. In a 2018 Drug Information Association white paper [28], regulatory perspectives from Center for Drug Evaluation and Research staff members note that understanding the etiology, natural history of the condition, and unique aspects of disease manifestation can help leverage what is known about the rare disease and inform measurement. A particular challenge that is not well addressed is the quantitative evaluation of a COA measure in rare populations. As pointed out in this commentary, content validity must be rigorously researched, documented, and measurement considerations for each concept planned carefully. In many circumstances, it will not be possible to conduct traditional psychometric validation in the relevant population. Under these circumstances, adapting existing measures and using item banks and generic measures with norms can all be creative substitutes for the traditional approaches. Qualitative interviews embedded in the clinical trial program can help inform responder thresholds that indicate a treatment response. Such qualitative data, of course, are not the only way to obtain meaningful change thresholds and would be secondary to traditional anchor-based approaches, and previously developed RDs using established methods, where applicable.

In the absence of a detailed regulatory guidance for the selection and validation of COA measures, it seems that the best regulatory strategy includes planning for early communication and collaboration among stakeholders, along with the flexibility to explore novel design and analytic strategies, while building on existing knowledge of COAs in general and HRQoL measures in particular. In a field with over 7000 unique diseases, the development of more specific regulatory guidance to clinical outcome selection and development may indeed not be feasible, but collaborative communication among key stakeholders may ensure that rare disease drug development programs capture the effect of treatments on how patients feel and function.

## Conclusions

In conclusion, we have elucidated many of the challenges associated with assessing HRQoL in rare diseases, practical barriers, possible solutions, and the regulatory context. In the absence of specific regulatory guidelines, creative application of general principles for design and validation must be relied on to fill the gap, including use of natural history studies, targeted literature reviews, clinical expert input, patient input, and adaptation of item banks, existing measures, and novel statistical approaches where possible. Quality of life may be the most important endpoint of all for many of these patients, and the challenges of assessing it in a valid way are worth the effort, and require innovative thinking suitable for each context.

## Data Availability

Not applicable.

## Abbreviations

COA: Clinical outcome assessment
FDA: Food and Drug Administration
HAE: Hereditary angioedema
HRQoL: Health-related quality of life
IRT: Item response theory
ISPOR: Professional Society for Pharmacoeconomics and Outcomes Research
MDRI: Multi-domain responder index
ObsRO: Observer-reported outcome
ODA: Orphan Drug Act
PD: Parkinson’s disease
PF: Physical functioning
PKAN: Pantothenate kinase-associated neurodegeneration
PRO: Patient-reported outcome
PROMIS: Patient Reported Outcome Measurement System
RD: Responder definition
TGCT: Tenosynovial giant cell tumor
UPDRS: Unified Parkinson’s Disease Rating Scale
US: United States
WHIM: Warts, hypogammaglobulinemia, infections, myelokathexis
